# Supplementary material for: Are carbon emissions trading and green financial instruments synergistic? -Comprehensive quantitative research based on content analysis
Source: PLoS One. 2024 Mar 7;19(3):e0298601. doi: 10.1371/journal.pone.0298601 (PMC10919601; doi:10.1371/journal.pone.0298601)
Supplement: S1 Data — (ZIP) [file pone.0298601.s001.zip › Data/地级市Z_F工作报告.zip!/║╙▒▒/▓╫╓▌╩╨/└┤╫╘╣1⁄2╓┌║┼í╛┬φ┐╦╩2╛▌═°í┐_2013.docx]

各位代表：

现在，我代表沧州市人民政府，向大会作工作报告，请予审议，并请各位政协委员和列席会议的同志提出意见。

一、过去五年的工作

2008年以来的五年，是我市改革发展进程中不平凡的五年。在省委、省政府和市委的正确领导下，我们坚持以科学发展观为指导，紧紧围绕建设沿海强市、和谐沧州目标，解放思想，锐意进取，持续不断地增投入、上项目、保增长、惠民生，全市经济社会实现平稳较快发展，圆满完成了十二届人大确定的各项任务目标。

——综合经济实力快速提升。五年来，我们积极应对国际金融危机等不利因素影响，顺利完成“十一五”规划，实现“十二五”良好开局，主要经济指标连续保持两位数增长。2012年，全市地区生产总值完成2811.9亿元，是2007年的1.78倍，年均增长12.3%，增速连续五年保持全省首位；全部财政收入完成380.4亿元，其中，公共预算收入142.6亿元，是2007年的3.07和3.02倍，年均分别增长25.2%和24.7%，全部财政收入连续四年居全省第三位；全社会固定资产投资完成1950.1亿元，五年累计完成6892.3亿元，年均增长25.8%。

——渤海新区开发建设取得重大突破。黄骅港综合港区一期工程建成通航，二期工程20万吨级航道、2个5万吨级液体化工泊位和4个10万吨级通用散杂货泊位开工建设，实现了从单一煤炭输出港向综合大港的转变。黄骅港吞吐量突破亿吨大关，2012年达到1.3亿吨，其中综合港区完成2000万吨，集装箱突破10万标箱。临港产业加速聚集，推进实施亿元以上重点项目400多个，总投资9400亿元。中铁装备新材料、中钢8万吨镍铁、沧州大化10万吨T DI建成投产，中海油中捷石化炼油质量升级改造、中国一重300万吨冷轧薄板、北汽40万辆整车等项目顺利推进，法液空、香港华润等一批世界和中国500强企业落户渤海新区。沧州临港经济技术开发区被批准为国家级开发区、国家循环化改造示范试点园区。大力推进基础和配套设施建设，实施了一批道路、供电、供水工程，航运大厦、港务大厦、联检办件大厅投入使用，黄骅新城建设取得重大进展。科技人才工作成效明显，引进国家级和省部级科研机构18家。2012年，渤海新区完成地区生产总值470.3亿元，全部财政收入88.3亿元，分别占全市的16.6%和23.2%。

——县域经济增势强劲。特色产业发展迅猛，园区建设成绩斐然，省级及以上开发区和工业聚集区达到19家，拥有主营业务收入超100亿元的产业集群7个。农业生产稳定增长，农林牧副渔总产值完成576亿元，增长4.2%，完成造林120万亩，森林覆盖率达到24%。五年新增国家级农业标准化示范区6个、农民专业合作社2058家、省级以上龙头企业21家，河间国欣、青县小洋人、泊头万雉园被评为国家级产业化龙头企业。认真落实各项惠农强农政策，累计发放粮食和农资直补46.96亿元。2012年，12个县、市（不含黄骅市和沧县）完成地区生产总值1814亿元，全部财政收入189.6亿元，分别比2007年增长76.2%和180.3%，占全市的63.9%和49.8%。全市所有县（市）财政收入均超3.5亿元，其中9个县（市）超10亿元。

——中心城区持续快速发展。以“三年大变样”为契机，全面加强中心城区规划、建设和管理，五年完成城市建设投资910亿元。沧州体育馆、规划馆、体育场、会展中心、政务服务中心、高铁站建成投用；金狮和阿尔卡迪亚两座五星级酒店建成运营；狮城公园、名人植物园、千童公园、运河景观带、文庙复建竣工开放；天成郡府、东方世纪城等精品住宅小区交工入住；维明路立交桥、沧州博物馆、图书馆、游泳馆、20家企业总部等重点工程扎实推进。五年新建、改扩建城市道路45条、63公里，新增天然气用户5.5万户、供热管网129公里、集中供热面积1250万平方米。新增、改造城市绿地面积763万平方米，建成区绿化覆盖率达到37%，成功创建省级园林城市。市区空气质量二级以上天数达到346天，比2007年增加34天。中心城区经济快速发展，华北商厦三期、红星美凯龙、居然之家等名品名店建成投用，泰大国际、锦绣天地等一批商业综合体加快推进。沧州高新区和沧东开发区跻身省级园区行列，沧东物流园区增列为省级物流聚集区，台商产业转移创新基地、航天神舟太阳能光热产业园等落地开工。2012年，中心城区完成地区生产总值554.2亿元，全部财政收入102.5亿元，分别是2007年的1.85倍和2.9倍，占全市的19.5%和26.9%。

——经济结构不断优化。工业经济持续稳定增长，2012年，规模以上工业实现增加值1145.2亿元，主营业务收入突破4000亿元，利税突破500亿元。“1+1”技改工程扎实推进，累计实施亿元以上技改项目631个，完成投资575亿元，新增规模以上工业企业622家，总数达到1774家，销售收入超百亿元企业达到6家。服 务业规模不断扩大，服务领域不断拓展，建成一批现代物流、商贸流通、文化旅游重点项目，三次产业结构调整为11.3:52.6:36.1。科技创新能力显著提高，香港南华、北京三友等一批科技型骨干企业落户沧州，国家级高新技术企业达到64家。新增“津桥”“十里香”等10个中国驰名商标，总数达到15个。深入实施“双三十”和“8755”减排工程，坚决淘汰落后产能，2012年万元G D P综合能耗比2007年下降25.18%，主要污染物排放总量低于省控目标。

——基础设施日益完善。邯黄铁路开工建设，京沪高铁建成运营，沿海高速、廊沧高速、大广高速建成通车，沧州环城高速全线贯通。新增铁路营运里程138公里、高速公路168公里，新改建国省干线267公里、农村道路4624公里。杨埕水库、东光观州湖水库建成使用。华润沧州 热电、黄骅和海兴风电并网发电，全市发电装机容量达到405万千瓦，比2007年增加220万千瓦。五年发展节水灌溉219万亩，维修桥、闸、涵685座，新建农村沼气池12.6万个，农村生产生活条件持续改善。

——改革开放成效显著。稳步推进国有工商企业改革，妥善处理职工安置等突出问题，成功组建沧州建设投资公司、河北渤海投资公司和沧州市公交集团。农村综合改革、财 税金融体制改革、医药卫生体制改革、文化体制改革有序推进。金融生态环境不断优化，华夏、浦发等6家股份制银行相继落户沧州，全市银行业金融机构达到19家。天交所渤海股权交易中心、河北恒银期货沧州营业部挂牌营业，境内外上市企业达到33家，实现上市企业县（市）全覆盖，连续四年被省政府授予“金融生态市”荣誉称号。对外开放步伐加快，与中石油、中石化、中钢、北汽等大型企业集团实现战略合作，香港联塑、韩国S K、美国O I等外资企业落户沧州，五年实际利用外资12.1亿美元，引进省外资金1270亿元。外贸出口持续扩大，2012年全市完成出口总值21亿美元，比2007年增加9.9亿美元。

——人民生活水平大幅提高。2012年，全市城镇居民人均可支配收入20805元，农民人均纯收入7514元，分别比2007年增加9422元和3449元，年均增长12.8%和13.1%。五年累计新增城镇就业24.7万人，下岗失业人员实现再就业12.4万人，农村劳动力转移就业70.9万人，城镇登记失业率控制在4.5%以内。开工建设保障性住房8.3万套，解决了2.4万户城镇低收入家庭住房困难问题，城市人均居住面积达到35平方米，比2007年增加4.71平方米。实施农村危房改造3.3万户，发放补贴资金1.7亿元。社会保障体系不断完善，城乡居民社会养老保险、基本医疗保险制度实现全覆盖。新型农村合作医疗稳步推进，住房公积金制度覆盖面逐年扩大。大力实施饮水安全工程，解决了286万人饮水安全问题。全面落实国家和省新十年扶贫开发纲要，五年累计投入扶贫开发资金13亿元，46万人口实现稳定脱贫。

——社会事业全面进步。五年新建、改扩建中小学校742所，消灭D级危房127万平方米，沧州师专成功升本。市中心医院、人民医院、中西医结合医院综合病房楼建成投用。新建、改扩建县级医院15个，政府办基层医疗机构全部实行基本药物零差率销售。成功举办第十二届、十三届中国吴桥国际杂技艺术节沧州会场、第八届中国沧州国际武术节和2012中国工笔画展，西路梆子、六合拳入选国家级非物质文化遗产名录。深入开展安全生产专项整治行动，强化食品药品安全监管，不断加大社会治安综合治理力度，严厉打击各种刑事犯罪，人民群众安全感普遍增强。积极推进“双拥”共建活动，连续三届获得“全国双拥模范城”荣誉称号，军政、军民团结的良好局面进一步巩固。统计、档案、人防、地震、民族宗教、人口计生、妇女儿童、残疾人等各项事业也都取得了新成绩。

——政府自身建设不断加强。深入开展“爱沧州、做贡献、干成事、出亮点”活动，大力转变工作作风，提高政府服务效能，风清气正、干事创业氛围日益浓厚。完善重大决策咨询、听证、对话制度，健全突发公共事件应急处置机制。加强机构编制管理，严格控制财政供养人员数量。认真落实党风廉政建设责任制，加大审计、监察工作力度，严肃查处违法违纪案件。严格依照法定权限和程序履行职责，主动向市人大、市政协报告工作、通报情况，五年共办理市人大代表建议583件、市政协提案1486件，代表委员满意率96.7%。

各位代表，奋斗历尽艰辛，成绩来之不易。过去的五年，是我们在科学发展观指引下，进一步理清发展思路，探索和开辟沧州科学发展新途径的五年；是我们抢抓机遇，发挥优势，推动经济社会又好又快发展，综合实力提升最快的五年；是我们顺应人民群众新期待，加大城市建设和民生投入，城乡面貌变化最大、人民群众得到实惠最多的五年。这些成绩的取得，是中共沧州市委正确领导的结果，是市人大、市政协鼎力支持的结果，是全市广大干部群众团结一心、砥砺奋进的结果。在此，我代表沧州市人民政府，向全市各族人民，向人大代表、政协委员和各民主党派、工商联、无党派人士，向离退休老干部、驻沧各单位、人民解放军和武警官兵，向所有关心、支持沧州发展的同志们、朋友们，致以崇高的敬意和衷心的感谢！

在总结成绩的同时，我们也清醒地认识到，发展不足依然是沧州最大的实际，公共预算收入偏低、中心城区辐射带动能力弱、城镇化水平不高仍是我们面临的突出矛盾；经济外向度低，与沿海临港区位优势还不相称；调结构、转方式、扩总量、提质量的任务十分艰巨；发展环境和生态环境有待进一步改善；一些干部的思想作风、工作能力与新形势、新任务的要求还不适应。这些矛盾和问题，我们将在今后工作中，下大力加以解决。

各位代表，回顾过去的五年，我们走过了一段不平凡的历程，跨过了一道道难关，创造了一个个奇迹。沧州人民在建设沿海强市的征程上，信心更加坚定，意志更加坚强，步伐更加坚实有力。

二、今后五年的总体要求和主要任务

今后五年，是深入实施“十二五”规划和《河北沿海地区发展规划》的重要时期，也是全面建成小康社会，推进沿海强市、和谐沧州建设的关键时期。

党的十八大提出到2020年全面建成小康社会目标，我国经济发展的基本面长期趋好；随着《河北沿海地区发展规划》、京津冀区域经济一体化、首都经济圈发展规划的加快推进，省委、省政府提出举全省之力打造渤海新区增长极，我市迎来多重叠加的机遇和优势；经济总量不断扩大，综合竞争力日益增强，为全市经济持续快速发展奠定了坚实基础。同时，我们也面临诸多风险和挑战，世界经济低速增长态势仍在延续，国内外环境不确定不稳定因素增多，宏观经济形势依然存在下行风险；环渤海地区加速发展，资源、要素竞争日趋激烈，转型升级、环境保护压力加大。对此，我们必须始终保持清醒的认识，既对未来充满信心，坚定发展决心，又要审时度势，沉着应对，不失时机地推进全市经济社会又好又快发展。

今后五年政府工作的总体要求是：坚持以邓小平理论、“三个代表”重要思想、科学发展观为指导，全面贯彻党的十八大精神，紧紧围绕建设沿海强市、和谐沧州目标，牢牢把握“稳中求快、稳中求好”主基调，以提高质量效益为中心，以转变发展方式为主线，以改革开放为动力，调结构、稳增长、优环境、惠民生，着力培育经济增长点，加快新型工业化、信息化、城镇化和农业现代化步伐，推动沿海经济、县域经济、中心城区经济协调发展，全力打造靓丽、繁华、宜居、和谐新沧州。

经济社会发展的主要目标是：

——综合经济实力全面提升。在提高质量和效益的基础上，经济总量再上一个新台阶。地区生产总值力争达到5000亿元，全部财政收入超过700亿元，率先实现全面建成小康社会目标。

——三大经济板块支撑能力持续增强。渤海新区地区生产总值力争达到1500亿元，全部财政收入260亿元，占全市的25%和30%以上；县域经济地区生产总值达到3000亿元，全部财政收入300亿元，占全市的55%和40%以上；中心城区地区生产总值达到1000亿元，全部财政收入200亿元，占全市的20%和25%以上。

——经济结构调整实现更大突破。现代农业扎实推进，新型工业化步伐加快，第三产业对经济发展的贡献率持续提高，三次产业结构更趋合理。高新技术产业、战略性新兴产业比重进一步提升，科技、人才支撑作用明显增强。

——生态环境质量明显提高。资源节约型、环境友好型社会建设取得重大进展，循环经济发展水平和能源资源利用效率不断提高，可持续发展能力显著增强。城市生活垃圾无害化和污水处理率达到100%，绿化覆盖率达到38%，人均公园绿地面积10平方米，力争所有县（市）进入国家或省级园林城市行列。

——社会事业和民生改善全面进步。教育、文化、体育、卫生等各项社会事业全面发展，城乡居民收入比2010年实现“双倍增”；社会就业更加充分，城镇登记失业率控制在4.5%以内；社会保障体系进一步完善，基本社会保障制度实现全覆盖；中低收入家庭住房保障体系基本建立；城市公共安全保障有力，社会安定有序、和谐进步。

实现上述要求和目标，突出抓好六大战略任务

（一）以渤海新区开发建设为龙头，大力发展沿海经济。举全市之力推进以渤海新区为龙头的沿海地区开发建设。充分发挥20万吨级航道作用，新建一批5-20万吨级矿石、原油、集装箱等系列码头群。加快高速公路、城际铁路、机场等集疏运体系建设，力争到2015年，黄骅港吞吐量达到3亿吨。培育壮大石油化工、冶金装备、电力能源、现代物流四大产业，打造具有国际影响力的临港产业聚集区、循环经济示范区。加快生态新城建设，促进港口、产业、新城协调发展。

（二）以加快转变发展方式为主线，推动产业结构优化升级。坚定不移地走新型工业化道路，促进工业化与信息化深度融合，增强产业核心竞争力。大力实施工业倍增计划，做强存量，做大增量，壮大提高石油化工、管道装备、机械制造等支柱产业，培育发展新能源、新材料、生物医药等战略性新兴产业。坚持拓宽领域、提升功能、创新业态，全面提高服务业发展水平。大力发展现代农业，着力构建集约化、专业化、组织化、社会化相结合的农业经营体系。

（三）以提高城镇化质量为重点，统筹城乡协调发展。优化城市功能定位和规划布局，走集约、智能、绿色、低碳的新型城镇化发展道路，构建以中心城市为依托，县城为支撑，小城镇和农村新型社区为基础，符合发展规律、具有沧州特色的城镇化发展格局。坚持“三城”统筹、协调推进，全面提高城市建设、管理和发展水平。抓住国家推进城镇化的有利时机，创新户籍管理体制机制，有序推进农村转移人口市民化。

（四）以深化改革开放为力，力营造经济发展新优势。坚持进一步解放思想，以创新思维、开放理念、市场的办法，破解体制机制制约，推进转型发展。继续深化行政管理体制改革，加快医药卫生体制改革，积极稳妥推进国有和集体企业改革、财税体制和投融资体制改革。发挥沿海临港、交通便利、土地资源丰富、发展低成本等综合优势，强化与央企、外企及大型民营企业对接合作，着力构筑面向京津，内引东南沿海和港口腹地，外联发达国家和地区的对外开放新格局。

（五）以“两个环境”建设为保障，不断提高可持续发展能力。积极推进邯黄、石港高速公路，石沧港、张保港铁路，谋划实施国道、省道升级改造，高速公路连接线及城区道路畅通工程，进一步完善现代综合交通路网格局。坚持引黄和引江、海水淡化相结合，全力做好引蓄水文章，重点推进南水北调中线及配套工程，积极开辟引黄线路，提高水资源保障能力。进一步削减行政审批事项，简化办事流程，提高办事效率，打造风清气正、开放文明的发展环境。加强生态环境保护，着力抓好减排、清水、净天、增绿工程，让沧州的天更蓝、水更清、地更绿、空气更清新。

（六）以保障和改善民生为目的，全面建设和谐沧州。坚持以人为本、民生优先，大力发展科技、教育、文化、体育、医疗卫生等各项社会事业。积极拓宽就业和创业渠道，推动实现更高质量的就业。加强城乡社会保障体系建设，持续提高统筹层次和保障水平。加强和创新社会管理，努力化解各种社会矛盾，全力维护社会和谐稳定，不断增强人民群众的幸福感和满意度。

各位代表，建设靓丽、繁华、宜居、和谐新沧州，既是全市人民的美好期盼，也是政府工作的目标和重点。我们已经打下了一个良好的发展基础，我们也一定能够谱写更加辉煌的新篇章！

三、2013年的重点工作和主要举措

今年是全面贯彻落实党的十八大精神的开局之年，是为全面建成小康社会奠定坚实基础的关键一年，做好今年的政府工作意义重大，影响深远。

综合考虑各方面因素，今年全市经济社会发展预期目标是：地区生产总值增长10%，全社会固定资产投资增长21%，全部财政收入和公共预算收入可比分别增长11%和12%，规模以上工业增加值增长14%，城镇居民人均可支配收入和农民人均纯收入增长10%。

（一）统筹三大经济板块，推动市域经济协调发展

1、全力打造渤海新区增长极。贯彻落实省委、省政府关于渤海新区开发建设的一系列重大决策部署，着力增强沿海经济的龙头带动作用，确保地区生产总值达到600亿元，全部财政收入超百亿元。重点抓好十项工作。一是抓港口建设。加快20万吨级航道和矿石码头建设，确保同步建成、同步使用。积极推进散杂货、液体化工等码头建设，确保14个泊位顺利开工。二是抓港口运营。加强码头运营管理，力争港口吞吐量达到1.7亿吨，集装箱突破20万标箱。加强通关设施建设，探索改善“大通关环境”，提高通关效率，力争黄骅港口岸正式开放。三是抓产业聚集。全力推进中国一重300万吨冷轧薄板、旭阳化工40万吨己内酰胺等99个亿元以上在建项目，确保北汽40万辆整车一期、鑫海石化等项目竣工投产。加快推进项目前期工作，确保佳龙芳烃联合装置等96个项目开工建设，力争渤海新区综合保税区获国家批准。四是抓招商引资。打好国家战略重点支撑发展区域、国家级开发区等金字招牌，加强宣传推介，着力引进战略投资者，提高招商引资实效。抓好在谈及签约外资项目落实，争取早落地、快开工。五是抓基础配套设施。加快沧东电厂三期、渤海新区热电以及供水、供气、输变电设施建设，推进企业总部、学校、医院、住宅和城区绿化等重点工程建设，进一步改善发展环境和生态环境。六是抓科技创新。加强与科研机构的战略合作，全面推进产学研一体化，力争引进省级以上重点实验室、技术研发中心总数达到30家。七是抓人才引进。落实人才特区优惠政策，引进500名以上各类专家和急需高端人才。八是抓体制创新。建立精简高效的管理机制，充分调动干部职工干事创业的积极性。九是抓融资保障。积极引进金融机构，创新金融产品，打造河北金融改革试验区。做大融资平台，力争可控资产突破500亿元。十是抓腹地开拓。加强与冀中南及广大腹地城市对接合作，发挥石家庄、邢台、德州、银川等地办事处作用，积极推进内陆港建设，形成港口和腹地互动发展新格局。

2、提升县域经济综合竞争力。坚定不移地实施工业强县战略，走产业立县、特色发展之路，推动县域经济扩规模、提效益、上水平，确保县域经济地区生产总值达到2000亿元，全部财政收入达到210亿元以上。一是发展壮大特色产业集群。立足产业基础，突出特色、品牌，挖掘增长潜力，引导生产要素向县域特色主导产业集中，打造一批立市兴县的龙头企业、“小巨人”企业，增强县域特色产业竞争力。重点抓好石油化工、管道管件、电线电缆、铸造模具、裘皮服装、五金机电等传统优势产业升级换代，发展壮大数控机床、功能材料、精细化工等新兴产业。二是全面提高县域经济开放水平。突出县域优势，全面推进对外开放，以资源换技术，以品牌换资金、换市场，吸引国内外知名大企业大集团落户沧州，通过合作发展，不断提高企业装备和管理水平，为县域经济发展提供动力支持。三是大力发展民营经济。全面落实促进民营经济发展的政策措施，放开经营领域，降低准入门槛，形成各种所有制经济平等竞争、相互促进的发展格局。实施民营企业“成长工程”，精心筛选一批有实力、成长性好的民营企业，组织优势资源，集中倾斜扶持，促其做大做强，让民营经济在县域经济中挑大梁、唱主角。四是推进县域经济晋级升位。深入开展对标行动，筛选确定标杆，全力对标赶超，实现高点定位、高速进位，力争各县（市）综合经济实力在全省排名明显提升。3、发展壮大中心城区经济。坚持一手抓服务业，一手抓工业，繁荣中心城区经济，提高辐射带动能力，确保中心城区地区生产总值超600亿元，全部财政收入力争达到110亿元。一是大力发展服务业。加快传统服务业改造升级，推进小南门、火车站、颐和广场等商业区建设。积极培育金融保险、现代物流、电子商务、信息中介等现代服务业，加快沧东物流产业聚集区、沧西物流产业带，及彩龙国际、荣盛国际等商业综合体建设，发展壮大总部经济、会展经济、城市夜经济，丰富发展内涵，拓展发展领域。二是大力发展新型工业。加快沧州开发区、沧州高新区、沧东开发区和新华、运河工业园建设，大力开展招商引资，引进建设一批新型工业化项目。积极推进台商产业转移创新基地、航天神舟太阳能、东塑新能源材料等重点建设项目，力促华油飞达石油钻机、那瑞化学中试基地、福道冶金轧辊等重点项目竣工投产。三是推动房地产业平稳健康运行。认真研究推动房地产业持续健康发展的对策措施，加快房地产项目建设。坚持廉租房、经济适用房等保障性住房和商品住房统筹推进，满足不同层次住房需求。

（二）培育七大经济增长点，着力构筑发展的有力支撑

1、抓重点项目建设。坚持产业项目、基础设施项目、民生项目一起抓，夯实发展基础，增强发展后劲。一是抓项目竣工投产。加快推进渤海重工、中航重机、邯黄铁路等一批在建重大项目，确保100个亿元以上项目竣工、投产。二是抓项目开工。加快推进任丘100万吨乙烯、运东华润热电、石钢搬迁等重点项目的前期工作，确保泰恒特钢40万吨铬铁、嘉好粮油大豆加工，以及京沪高速沧州市至冀鲁界段，石津干渠、保沧干渠等200个亿元以上重点项目开工建设。三是抓重大战略支撑项目。加快华北石化炼油质量升级改造、金牛化工40万吨P V C等30个重大战略支撑项目建设，及时协调解决资金、用地等突出问题，确保按要求如期推进。四是抓项目谋划储备。加大谋划力度，争取更多项目列入国家和省重点建设计划，确保市级储备亿元以上项目超过1000个。

2、抓工业经济。坚持科技引领、创新驱动，深入开展企业“对标”行动，推动工业经济转型升级。一是做大做强优势骨干企业。以“1+1”工程为抓手，大力实施企业“登台阶”计划，重点抓好沧州大化5万吨T D I技术改造、鑫宜达钢管等190个技改扩规项目，推动企业技术进步、产品升级，打造一批在全省、全国有重大影响的大企业大集团，确保新增销售收入超50亿元企业6家、100亿元企业1家，总数分别达到15家和7家。完善扶持政策，加大企业上市推进力度，引导鼓励企业到境内外多层次资本市场上市融资，确保每县（市、区）至少新增1家上市企业。二是推进中小微型企业晋级升位。认真落实扶持中小微型企业发展的政策措施，着力解决企业融资、用地、项目审批等突出问题，推动个体工商户向小微企业转变，小微企业向规模以上企业发展，力争年内新增规模以上工业企业200家。三是下大力抓好节能减排。坚决淘汰落后产能，鼓励企业采用先进设备、技术和工艺，提高资源、能源利用效率。大力发展循环经济，以创建生态示范园区、示范企业为抓手，积极推行清洁生产，减少污染和废弃物的排放，实现企业有机衔接、产业循环发展。

3、抓城镇化建设。统筹推进城市建设、城市经济和城市管理，全面提高新型城镇化水平。一是加快中心城区重点城建工程建设。以西部城区、东部新城、火车站周边旧城区、运河景观带“三区一带”为重点，以十大城建工程为抓手，推进东西部城区均衡发展，努力打造美丽沧州。园林绿化工程：大力实施绿色行动三年计划，加快滨河公园、体育公园、文化公园等9个公园建设，新建街头游园10个。着力抓好永济西路、广州路、新疆大道等11条道路绿化，沧州师院、沧州炼油厂等196个社会绿化项目。推进环城林带建设，新增造林面积9680亩。道路畅通工程：新建青海大道、纬三东路、海丰大道3条道路，加快维明路地道桥及引道工程、广州路、上海路等续建工程建设，着力抓好流津时代、嘉禾一方等12条小区配套道路建设。开发区、高新区通道建设工程：积极推进开发区兴业路西延、石黄高速沧州东互通连接线、西绕城二期工程，打通永安大道，拓展开发区、高新区与市区的连接通道。市政设施配套工程：重点抓好鞠官屯、育红路等地雨水、污水泵站及配套管网建设，实施运东、运西污水联网工程；新建燃气中低压管网45公里，供电线路30公里，集中供热主管网3.4公里；新建5个环卫垃圾处理设施项目。东部集中供热工程：积极推进运东集中供热工程，开工建设一期两台100吨位锅炉项目。“两改”工程：加大城中村和旧城区改造力度，启动实施大杨庄、华元一世界等城中村和旧城区改造项目，积极稳妥地做好居民回迁安置工作。精品住宅小区建设工程：加快皇家壹里、泰和世家等10个精品住宅小区建设。企业总部工程：加速管业大厦、传媒大厦等20个企业总部建设，确保如期竣工投用。商贸物流建设工程：加快恒顺世纪中心、明珠商贸城、台湾精品城等9个商贸物流项目建设，进一步拓展中心城区服务业。大型公建设施工程：加快沧州游泳馆、河北工专新校区、市医院医专院区等4个续建项目，开工建设新城消防指挥中心、人防宣传教育和技能培训基地，改造妇女儿童活动中心、青少年宫。二是加强城市管理。坚持建管并重、综合治理，加强基础设施和公共服务设施建设管理，深入开展城市环境综合治理，打造优美舒适、规范有序、便捷畅通、文明和谐的城市环境。三是积极推进县城建设。坚持把县城建设放在更加突出的位置，进一步提升规划、建设和管理水平，推进县城做大规模、完善功能、提升品位、打造特色。重点谋划实施好城市广场、公园、文化体育场馆、商品住宅等精品工程。四是统筹城乡一体化发展。坚持城镇化发展与新农村建设相结合，加快中心镇、产业镇和农村新民居规划建设，整治优化城乡街道环境卫生，推进道路交通、供排水、信息网络等基础设施和公共服务设施向城镇覆盖，向农村延伸。

4、抓服务业发展。认真落实服务业拓展实施方案，着力提高服务业发展水平，确保服务业增加值增长13%以上。一是改造提升传统服务业。依托中心城区和县城，加快商贸服务业改造提升步伐，健全城乡现代流通网络，建设一批大型综合批发市场和商业综合体，打造辐射周边区域的商贸服务中心。二是大力发展现代服务业。制定完善物流产业发展规划，研究出台更加优惠的政策，吸引一批大型物流企业集团落户沧州。加快物流产业集聚区、物流公共信息和大宗商品交易平台建设，构建以港口为龙头的综合物流体系，打造现代物流产业基地。加快中心城区中央商务区、企业总部基地、中介服务体系建设，积极引进国内外企业集团总部、贸易和通讯中介服务机构，鼓励发展担保、监理、咨询、评估、会计、律师服务业，建设区域性商务服务中心、信息服务中心。三是加快发展金融业。进一步优化金融生态环境，引导金融机构增强支持实体经济发展的主动性，创新产品、服务和管理，全面提升信贷资产规模和质量。加快建设沧州市企业融资服务中心，推进银企对接经常化、域外金融机构引进常态化，构建完善的金融业服务体系。四是积极培育新兴服务业。加大政策引导和扶持力度，积极发展文化体育、休闲健身、观光旅游、教育培训、社区服务和养老服务等新兴产业，打造休闲旅游度假基地和消费服务中心。

5、抓开发区和工业聚集区建设。坚持把开发区和工业聚集区建设作为科学发展的重要抓手，推进工业经济集约集群发展。一是编制完善园区发展规划。委托一流规划设计院所，高起点、高水平编制完善园区发展总体规划、控制性详规和产业发展规划，搞好规划环评，进一步提升园区科学发展的水平。二是加强园区基础设施建设。按照适度超前的原则，着力推进水、电、路、气、讯等基础设施建设，完善仓储物流、科技研发等服务配套设施，满足企业生产和发展需要。三是着力提升园区发展规模和层次。建立完善园区发展考核奖惩激励机制，创新管理体制，增强园区发展的内生动力。加强招商引资和项目建设，积极引进一批高技术、高效益、低能耗的龙头型、基地型项目，壮大园区经济规模，提升质量和效益。确保工业增加值、固定资产投资、税收等主要经济指标增速达到25%以上，沧州临港经济技术开发区、任丘开发区主营业务收入超过500亿元，沧州开发区、盐山工业聚集区等5家超200亿元。

6、抓农业农村经济。强化农业基础地位，加快农业规模化、标准化、集约化发展，促进农业增效、农民增收。一是积极推进农业产业化经营。落实扶持政策，加大支持力度，培育壮大一批规模大、带动能力强的龙头企业，打造一批知名农产品品牌，力争市级以上农业产业化龙头企业达到500家，新增农民专业合作组织500家。加快土地承包经营权流转，积极发展多种形式的适度规模经营。二是稳定发展粮食生产。认真落实惠农补贴政策，加强粮食核心区建设，力争粮食总产达到460万吨。三是大力发展特色农业。加大农业科技推广应用，大力调整和优化农业产业结构，建立一批规模大、集约化程度高的养殖小区，蔬菜、果品和优质棉花种植基地。大力支持近郊农业产业园建设，以果岭湾农业产业园、五针松观赏园为带动，建成一批集生产、观光、休闲于一体的城市后花园。四是加强农村基础设施建设。加大投入力度，完善基础设施，提高农业综合生产能力。积极推进以任丘、河间、献县、肃宁水库为重点的“十百千”工程，加强农田水利基本建设，发展节水灌溉30万亩。大力实施农村公路和电网升级改造工程，新建改建农村道路600公里，新增输电线路409公里。

7、抓对内对外开放。充分发挥我市综合优势，加大宣传推介和招商引资力度，引进资金、项目、人才，为经济发展增添新的活力。一是进一步增强沿海开放意识。牢固树立要实现率先发展就要率先开放理念，打好沿海临港牌、开放牌，积极主动对接沿海、融入沿海，充分利用国际国内“两个市场、两种资源”，实现借力借势发展。二是狠抓招商引资。突出招商重点，瞄准京津、长三角、珠三角，以及港台、日韩、欧美等国家和地区，抓住百家央企、民企、外企、科研院所进河北的机遇，加强对接合作，力争在引进世界500强、中国500强等战略投资者上实现重大突破。进一步创新招商方式，突出产业链招商、园区招商、以商招商，精心组织好各类招商引资活动，切实提高招商引资的针对性。进一步突出招商实效，加强与外国驻华商务机构、企业总部、行业协会、商会的联系沟通，研究跟踪产业转移投资走向，紧盯意向及签约大项目，以良好的发展环境、优质的合作项目吸引客商、聚集产业，确保全年直接利用外资突破4亿美元。三是扩大外贸出口。加强石油钻采设备、管道装备、精细化工、裘皮服装等出口基地建设，加大对重点出口企业扶持力度，引导企业巩固传统市场，开辟新兴市场，稳步扩大外贸出口规模。

（三）统筹发展社会各项事业，切实保障和改善民生

1、进一步提高社会保障水平。积极推进“五险”扩面提升，提高城乡居民医疗补助标准、报销水平和城乡低保标准，城乡居民养老保险参保率达到93%以上。全面推行乡村卫生服务一体化管理，积极推进乡镇卫生院、村卫生室建设，扎实做好县级公立医院综合改革。加强基层医疗卫生机构人才引进和培养，提升基层综合服务能力和水平。

2、大力发展社会事业。着力抓好学前教育改革试点工作，进一步提高普及率和优质率。均衡发展义务教育，提高教育教学质量。加快高中教育普及提升，努力办好职业教育，推动高等教育内涵式发展。坚持一手抓文体事业、一手抓文体产业，加快建设文化强市、体育名市。健全公共文化服务体系，推进全民健身活动开展，积极做好省十四届运动会筹备工作。充分发掘杂技、武术等优势资源，突出沿海特色、运河文化，努力打造文化精品。

3、抓好安全生产和食品药品安全。严格落实安全生产责任制，健全完善监督监察机制，重点抓好危险化学品、烟花爆竹、道路交通、建筑施工、人员密集场所等行业（领域）的安全监管，加大行政执法力度，确保不发生重特大安全生产事故。深入开展食品药品质量安全专项整治活动，加大生产、流通、销售等各个环节的监督检查，严厉惩处违法违规行为，保证人民群众饮食用药安全。

4、加强和创新社会管理。建立畅通社情民意沟通渠道，维护人民合法权益，解决群众合理诉求。加强城市社区建设，提高公共服务水平。进一步强化社会治安综合治理，依法防范和打击各类违法犯罪，确保社会和谐稳定。

今年重点实施十大民生工程：就业促进工程：全年新增城镇就业5.5万人，其中下岗失业人员再就业1.98万人，就业困难人员就业7200人，转移农村劳动力4.8万人。保障性安居工程：开工建设保障性住房26200套，竣工26000套，分配入住21500套。教育基础设施提升工程：加快沧州一中新校区、二中学生公寓、十六中及市区部分小学新建扩建工程，完成中小学校舍安全工程及7所农村初中和53所农村薄弱学校改造任务。“五险合一”服务平台建设工程：整合养老、医疗、工伤、失业、生育“五险”信息管理系统，建立统一的社会保险管理信息服务平台，方便群众办事，提高服务水平。医疗卫生服务保障工程：新农合筹资标准提高到每人340元，最高支付金额提高到9万元，参合率稳定在95%以上，政策范围内住院费用报销比例达到75%以上，积极做好20种重大疾病保障试点工作。扶贫开发工程：创新扶贫投入机制，加大产业扶贫力度，确保8万贫困人口实现稳定脱贫。农村集中饮水安全工程：新建联村集中供水水厂24个，续建68个，切实解决680个村、80万人的饮水安全问题。养老服务体系建设工程：强化光荣院、敬老院、福利院建设，扶持发展社会办养老服务机构，新建社区养老服务站40家，新增床位1200张，积极推进农村养老“幸福工程”，新建农村互助幸福院600个；全面实施80岁以上老人生活补贴制度。文体惠民工程：加强城乡公共文化、体育健身工程建设，完成170个乡镇综合文化站和582个帮扶村农家书屋、体育健身工程建设任务，市、县大型公共文化设施全部对社会免费开放。平安沧州创建工程：大力推行“一村一警、三支队伍”警务机制，每个行政村配备1名警务辅助人员，建立巡防、信息、民调三支队伍，提升农村地区治安防控的整体水平。推进“天网”和“路防”工程建设，布建科技“警务站”1664个，新建省界高清监控点15处、市区视频监控点860处，实现全市主要路口、重点部位视频监控全覆盖。

四、加强政府自身建设

新形势、新任务对政府工作提出了新要求，各级政府和全体公务人员要以深入开展“爱沧州、做贡献、干成事、出亮点”活动为动力，大力改进工作作风，加强政府自身建设，努力打造“为民、务实、清廉”政府。

一是进一步增强学习观念，不断提高攻坚克难能力。积极倡导全员学习，大兴调查研究之风，认真学习、深刻领会党的十八大精神、中央和省一系列重大决策部署，坚持与沧州实际相结合，切实把学习调研成果转化为谋划工作的思路、推进发展的举措、化解矛盾的本领，全面提高政府工作的预见性、针对性、创造性。

二是进一步增强效能观念，不断提高政府执行能力。大力弘扬雷厉风行、真抓实干的优良作风，高标准、快节奏抓好各项工作落实。坚持治庸、治懒、治散，强化目标责任管理，优化办事流程，提高工作效率。坚持少开会、开短会，深入基层一线，解决实际问题，干实事、办好事。

三是进一步增强法治观念，不断提高依法行政能力。坚持依法执政，严格按照法定权限和法定程序行使权力、履行职责。健全重大事项专家咨询、社会公示、听证质询制度，切实做到科学决策、民主决策、依法决策。自觉接受市人大及其常委会的法律监督，市政协的民主监督，广泛听取各民主党派及社会各界人士的意见和建议，不断改进政府工作。

四是进一步增强群众观念，不断提高为民服务能力。牢固树立以人为本、执政为民理念，多谋民生之利、多解百姓之忧、多办群众期盼之事。进一步强化服务意识，增强公共服务能力，提高公共服务效率，维护社会公平正义。进一步强化诚信意识，说了算、定了干，言必信、行必果，着力提升政府公信力。加大政务信息公开力度，充分保障人民群众的知情权、参与权和监督权。

五是进一步增强廉政观念，不断提高拒腐防变能力。加强惩治和预防腐败体系建设，管好钱，用好权，让权力在阳光下运行，切实做到干部清正、政府清廉、政治清明。严格执行中央关于改进工作作风、密切联系群众的八项规定，厉行勤俭节约，反对奢侈浪费，严格控制“三公”经费，把更多的财力用到经济建设和改善民生上，让人民满意，让群众放心。

各位代表，回首过去，沧州人民日夜期盼的大港梦、产业梦已经变成了现实，书写了光辉灿烂的历史；展望未来，沧州人民的强市梦、宜居梦正在一步步向我们走来，在新的征途上将谱写更加壮丽的宏伟篇章。让我们在省委、省政府和市委的坚强领导下，解放思想，开拓创新，求真务实，拼搏奉献，为加快建设靓丽沧州、繁华沧州、宜居沧州、和谐沧州而努力奋斗！
